# Exploring Population Pharmacokinetic Modeling with Resampling Visualization

**DOI:** 10.1155/2014/585687

**Published:** 2014-05-04

**Authors:** Fenghua Zuo, Jun Li, Xiaoyong Sun

**Affiliations:** ^1^College of Information Engineering, Taishan Medical University, Taian, Shandong 271016, China; ^2^Faculté de Pharmacie, Université de Montréal, CP 6128, Succursale Centre-Ville, Montréal , QC, Canada H3C 3J7; ^3^Centre de Recherche Mathématiques, Université de Montréal, CP 6128, Succursale Centre-Ville, Montréal , QC, Canada H3C 3J7; ^4^Agricultural Big Data Research Center, College of Information Science and Engineering, Shandong Agricultural University, Taian, Shandong 271018, China

## Abstract

*Background*. In the last decade, population pharmacokinetic (PopPK) modeling has spread its influence in the whole process of drug research and development. While targeting the construction of the dose-concentration of a drug based on a population of patients, it shows great flexibility in dealing with sparse samplings and unbalanced designs. The resampling approach has been considered an important statistical tool to assist in PopPK model validation by measuring the uncertainty of parameter estimates and evaluating the influence of individuals. *Methods*. The current work describes a graphical diagnostic approach for PopPK models by visualizing resampling statistics, such as case deletion and bootstrap. To examine resampling statistics, we adapted visual methods from multivariate analysis, parallel coordinate plots, and multidimensional scaling. *Results*. Multiple models were fitted, the information of parameter estimates and diagnostics were extracted, and the results were visualized. With careful scaling, the dependencies between different statistics are revealed. Using typical examples, the approach proved to have great capacity to identify influential outliers from the statistical perspective, which deserves special attention in a dosing regimen. *Discussion*. By combining static graphics with interactive graphics, we are able to explore the multidimensional data from an integrated and systematic perspective. Complementary to current approaches, our proposed method provides a new way for PopPK modeling analysis.

## 1. Introduction

Graphics is an important tool in data diagnostics that can be used to detect patterns, screen outliers, and test hypotheses [1–5]. In the field of pharmacokinetics, we can observe many elegant graphics used to help answer various biological questions. In his comprehensive tutorial book, Ette gave many examples of applying statistical graphics for the problems in pharmacokinetics and pharmacodynamics [2]. With detailed explanations of each plot type, he systematically reviewed their applications and pointed out that “the use of graphic techniques in data visualization aids understanding of the data structure that would lead to an informative data analysis.” At the same time, Karlsson described, from a model perspective, 22 assumptions for various situations during model development and then demonstrated the advantage of graphics in the assumption testing of the population pharmacokinetic (PopPK) model [6]. In addition, a detailed demonstration was given by Bonate, with real examples and diverse plots, showing how the graphics can greatly facilitate and impact each stage of the PopPK model-building process [7]. Our team developed a graphic pipeline to generate a collection of plots and statistics for PopPK model diagnostics. By automatically generating R scripts for diverse plots, we introduced the graphic flexibility and diagnostic efficiency for PopPK model building [8].

Resampling techniques, based on the intensive computational capacity of computers, have been widely applied to PopPK modeling for the assessment of uncertainty in parameter estimations and the detection of influential observations [9–12]. Though hundreds or thousands of data sets are produced with resampling, users generally tend to examine the numerical rather than the graphical summaries. However, considering the complex algorithms used in PopPK model fitting and the nonlinearity carried by the model, visualization of resampling statistics can be an efficient and direct way to gain a deeper understanding of the relationship between the model and the data.

In this paper, we develop a graphic approach to visualize resampling statistics using static and interactive graphics. The static plots are efficiently and conveniently connected through interactive graphics, which can help in exploring the associations between complex statistics and making discoveries. To our knowledge, no previous study has applied graphics, as in our approach, to explore and analyze the resampling data sets which are crucial in PopPK modeling. This is the first attempt in the pharmacokinetic field to incorporate interactive graphics in resampling data analysis. The current paper is organized as follows.  Section 2 explains the related graphic methods.  Section 3 reports the results for this research. The discussion and conclusions are combined in Section 4.

## 2. Statistical Graphics and Methods

### 2.1. Overview of Resampling Statistics

Resampling statistics is a term used to describe the statistical methods that take multiple samples from a data set and calculate quantities based on the estimations obtained from each new set of data. These methods include case deletion, which can be used to detect the outliers with substantial influence on the model fit, and the bootstrap, which helps measure the uncertainty associated with parameter estimates.

In PopPK, the data of different subjects may have different impacts on the modeling process. The lack in uniformity of several patients, that is, the outliers identified statistically, can seriously affect the model fit and parameter estimation and thus greatly degrade the explanation of the final model to the data. The case deletion methods remove all the observations related to a subject and refit the model with the reduced data set. The subject is considered to be influential if the new estimation of the parameters changes substantially, which are at the extremes or not within 5–95 percentiles of the confidence interval. To obtain comprehensive results, this process is generally repeated for each subject in the original data set. Some methods allow the deletion of groups of subjects, which may reveal the group effects on the final model fit. Simultaneously, in some circumstances, single observation may be deleted and single influential data points can be examined in detail.

The bootstrap is another sampling strategy with replacement [13]. We performed a few hundreds or thousands of bootstrap runs to obtain some robust results. By grouping some of these bootstrap samples and refitting the models, the users can extensively explore the distribution of parameter estimates. This method is typically used to add error bands or confidence intervals for parameter estimates.

**Figure 1:**
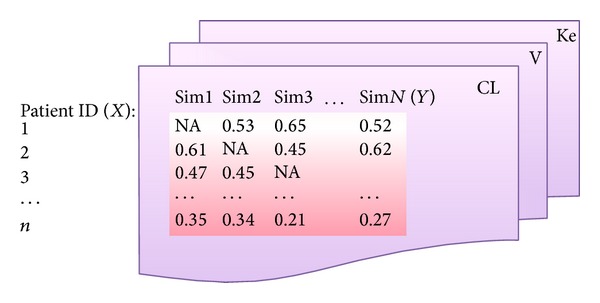
Multiple simulated data sets for resampling statistics. In the first simulation data set (sim1), when the subject is absent from the simulation, no parameter is estimated, so a missing value (NA) is generated. One of these tables is generated for each parameter (CL,* V*, and Ke). We can consider this to be a multivariate data set, which we will use to examine the influence of each subject and assess the variability in the parameter estimates.

Figure 1 illustrates the data structure of the final results of all interested pharmacokinetic parameters, which were calculated by creating multiple simulated data sets and refitting models with resampling data.

### 2.2. Graphic Methods

**Figure 2:**
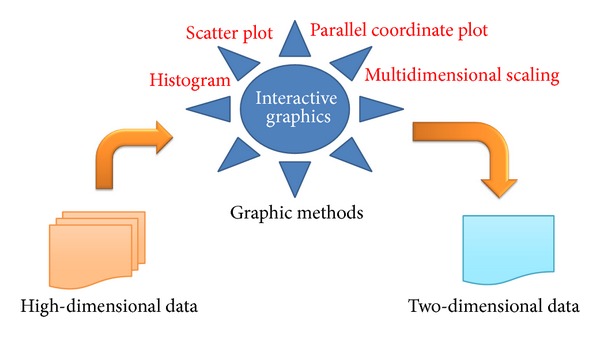
Graphic methods for visualizing resampling data.

Basic plots, such as histograms and scatter plots, are presented to explore the sample distribution and detect the influential objects (Figure 2). To examine the multivariate matrix, two well-known static graphical approaches, namely, specialized parallel coordinate plots and multidimensional scaling, were applied to the resampling data. All the methods can be implemented in R package* PKgraph* (http://cran.r-project.org/web/packages/PKgraph) [14]. The PKgraph functions as the interface to integrate all graphical tools for the diagnostic purpose. Specifically, the R packages* lattice* [15] and* ggplot2* [16] were used to produce the static graphics and* rggobi* [17] for the interactive graphics.

#### 2.2.1. Static Graphics

*Parallel Coordinate Plot*. Parallel coordinate plot was developed by Inselberg [18] and Wegman [19] to visualize an orthogonal axis system (Inselberg 1985, Wegman 1986). In this paper, this method converts a high-dimensional space to a two-dimensional one. The analysis steps are as follows.

1. A set of horizontal parallel axes were created from the columns of the data matrix. Each axis represents one data set, matching each case deletion run in this research.
2. Along each parallel axis, the empirical Bayesian estimates of a parameter (CL in Figure 6) were annotated for each subject. For a fair comparison among case deletion runs, all values were normalized by the difference between the maximum and minimum values of this run. To facilitate the visualization, two boundary lines, maximum and minimum, were set at the boundary. As such, in Figure 6, all case deletion runs have the same unit length: 1.0 (global maximum) − 0.0 (global minimum) = 1, which arranges all case deletion runs in the same scale.
3. The values for each subject were connected downwards by lines.

The final results were a set of plots that showed how the parameter estimates varied across all runs. If the estimate changed substantially, the subject deleted in that run was influential to the model fit.

*Multidimensional Scaling*. Multidimensional scaling (MDS) is a well-known strategy that transforms high-dimensional data into a low-dimensional representation, while preserving the relative distances. The main algorithm focuses on minimizing a loss function, which measures the difference between the distances in the high-dimensional space and the low-dimensional space. The final plot is generated from the low-dimensional data. There are many possible loss functions for MDS, including classical, metric, and nonmetric scaling. In this project, we used “Torgerson” loss function, which is the standard approach in R function “cmdscale.”

When the Euclidean distance between the points is used, MDS performs like principal component analysis, which has been proven to be a valuable tool in PopPK modeling, to identify the influential cases and delineate the variability [7, 20]. In addition, the MDS framework provides much flexibility in the construction of low-dimensional representations. A different distance metric and loss function can always be chosen to allow nonlinear mapping from a high-dimensional space to a low-dimensional one.

In this research, we used classical MDS to obtain a linear projection of the resampling statistics data and each simulation was considered as one dimension. In this way, MDS functions can be considered as a tool to summarize the variability across all simulations. As a reminder, each point corresponds to one subject in an MDS plot.

#### 2.2.2. Interactive Graphics

Interactive graphics is based on static graphics, and the users can make changes to the plots conveniently by simple mouse action. By linking the plots together, this technique enables the element changed in one plot to propagate to all other visible plots [21–24]. In this paper, we describe a graphic approach to visualize the resampling data by linking a histogram, a scatter plot, a parallel coordinate plot, and an MDS plot through interactive graphics.

### 2.3. Data

One data set from PKgraph [14] was utilized to illustrate the visualization approach. This data set has 100 patients, and each individual was sampled at 0, 0.25, 0.5, 0.75, 1, 1.5, 2, 2.5, 3, 4, 6, 8, 12, 16, 20, and 24 hours after dose. Weight (WT) was measured as a covariate for each subject.

The resampling statistics were generated and fitted using the* cdd* and* bootstrap* functions in PsN [25], and the results were analyzed with PKgraph. In each bootstrap, 100 patients were resampled and we repeated this process 50 times.

### 2.4. Model

A one compartment model of i.v. bolus administration was chosen to model the data set of drug concentrations. Its mathematical description is as follows:

(1) $$\begin{array}{c} C_{ij}=\frac{\text{Dose}}{V_{i}}e^{-(\text{C}{\text{L}}_{i}/V_{i})t_{ij}} \end{array}$$

for patients *i* = 1,…, *n* and samplings *j* = 1,…, *k*_*i*_. The standard exponential between subject variabilities for pharmacokinetic parameters as well as the combined proportional and additive error model was used. All data were fitted with NONMEM using the algorithm of first-order conditional estimation method with interaction.

## 3. Results

### 3.1. Resampling Design

Generally the resampling design needs to be validated. In the case deletion methods, each subject was subsequently deleted to detect the graphical patterns. For bootstrap, we would expect the distribution of subjects picked for deletion to be fairly uniform.

**Figure 3:**
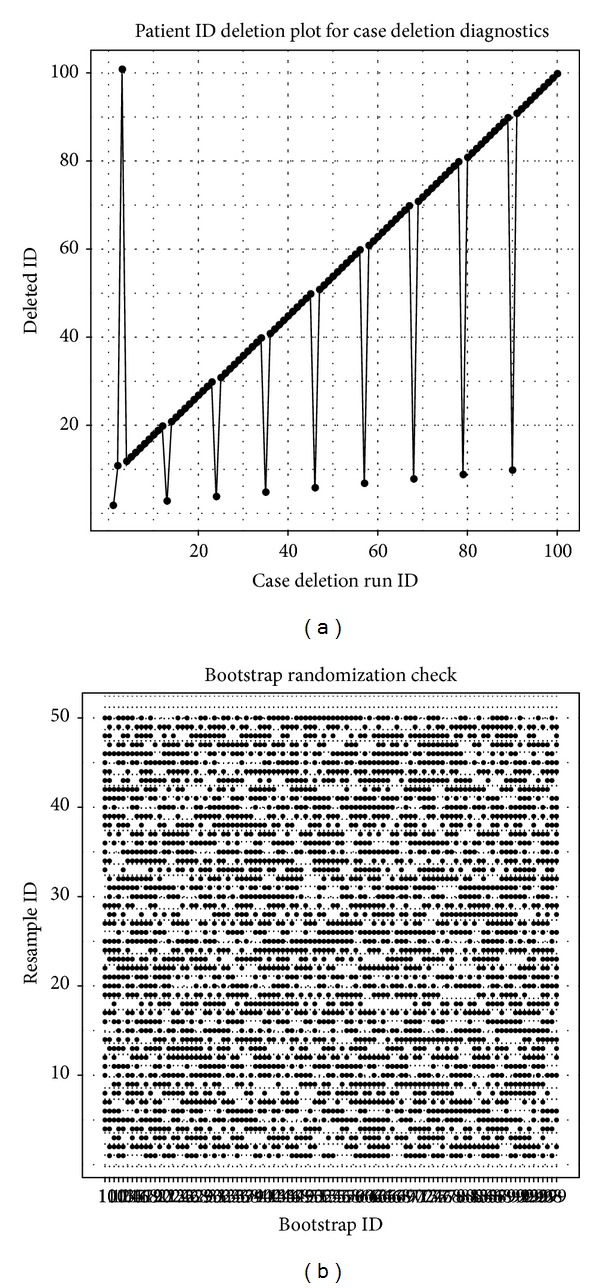
Plots to examine the resampling designs (a) for case deletion and (b) bootstrap. In each case the run ID is plotted horizontally, and the vertical axis displays which cases were in or out of the sample. For the case deletion design we plot the ID number of the subject deleted for the run. Lines connect sequential runs. If the deletion was done sequentially by PsN, then we would see a straight line from (1, 1) to (100, 100). This is not the case: in the first run, the first patient was deleted; in the second run, the 10th patient was deleted; and in the third run, the 100th patient was deleted.

Figure 3 shows the plots for the resampling design. Figure 3(a) is for case deletion diagnostics, and the subjects were not subsequently deleted according to their identification numbers for analysis. The process was described in the PsN documentation as a “perturb” pool. Most subjects were deleted sequentially, but the pattern was periodically broken by selecting one subject from the “perturb” pool.

Figure 3(b) is for the bootstrap. A dot point indicates a subject selected for the sample. It is clear that these dot points are uniformly distributed over the square, thus confirming the true randomness of the samplings. If the resampling with replacement is not random, the sample ID in the* y*-axis will be shown as a horizontal black or blank line in the plot.

### 3.2. Distribution of Demographic Covariates in Resampling Data

**Figure 4:**
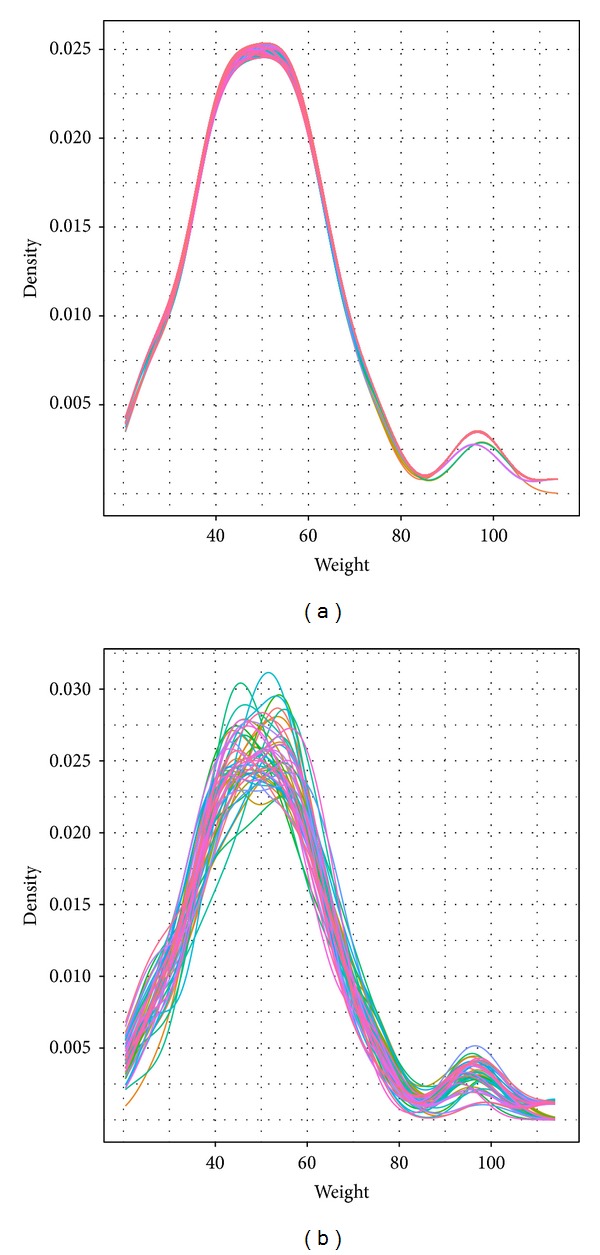
Distribution of weight for the samples: (a) case deletion statistics and (b) bootstrap. Each sample is represented using a different color.

Ideally, the resampling design is independent of the covariates used in the model fitting. To check this, we explored the distribution of the covariate for each simulation.  Figure 4 shows the density plots for weight (kg). It reveals that the subjects in the study were typically around 50 kg, with some overweight subjects at around 95 kg.

Figure 4(a) is a density plot for the case deletion statistics, where each simulation (one subject removed) is represented by a different color. Figure 4(b) explains the weight distribution for each bootstrap sample. We can observe that the weight distribution has a similar pattern for all samples in the former, while there is much more variation in the latter. Since some runs only included light-weight patients, this results in a skewed distribution. Simultaneously, a formal statistical test for equality of distributions is suggested to be performed to confirm this finding.

### 3.3. Distribution of Some Parameters in Resampling Data

**Figure 5:**
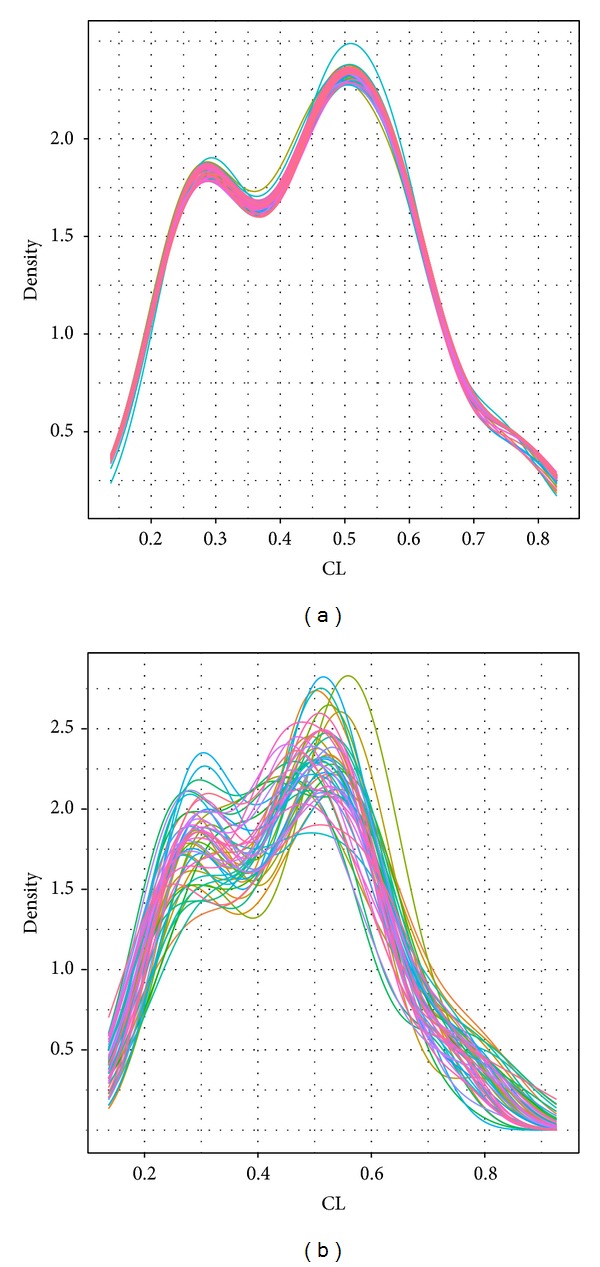
Distribution of clearance (CL) in (a) case deletion statistics and (b) bootstrap samples. There is more variability in the bootstrap samples, as expected. In the case deletion samples, one run had a noticeably higher second peak than those in other samples.

The basic properties of the model are determined by the estimates of certain model parameters, such as clearance (CL) and volume of distribution (*V*). It is of great value to examine the density of these estimates across samples for model diagnostics. Although population level parameters and intersubject variability are the important parameters too, for demonstration purpose we only chose CL and* V* to explore the visual diagnostic methods.  Figure 5 explores the distribution of clearance for each sample. Interestingly, the basic shape is bimodal, which means that there are two groups of clearances, around 0.275 and 0.5, respectively. Figure 5(a) shows the results from case deletion statistics, and the distribution of clearance is clearly similar for most samples. Figure 5(b), on the other hand, shows the distribution of clearance in the bootstrap samples. In particular, more variability was observed than in the case deletion samples, though no individual sample was found to be substantially different from the others. A statistical test is suggested to confirm this result.

### 3.4. Parallel Coordinate Plot and Multidimensional Scaling for Case Deletion Diagnostics

**Figure 6:**
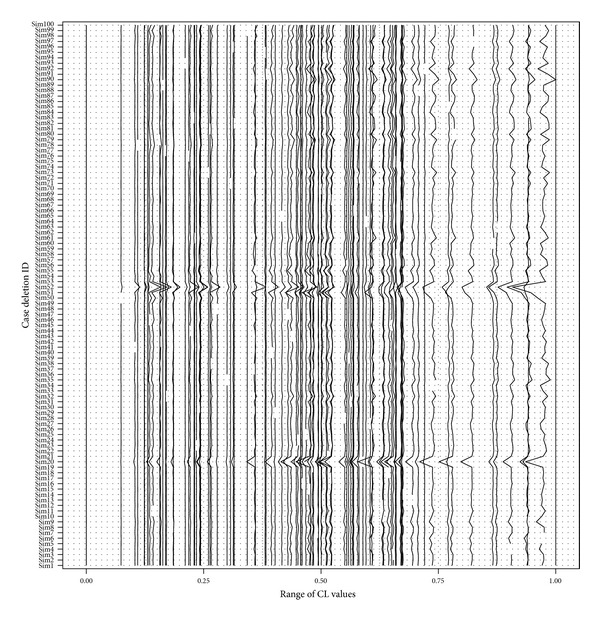
Parallel coordinate plot for diagnosing case deletion runs for the clearance estimates. Each line in the figure connects values for each subject, across runs. The clearance estimates change substantially for two runs, suggesting there are two patients which have undue influence on the model fit.

Figure 6 is a parallel coordinate plot for the scaled estimates of clearance from each model fit. The values inferred from each simulation were connected by subjects, and different colors were used to represent subjects. Interestingly, when two samples (subjects 52 and 20) were deleted, the estimated clearance changed substantially. Generally, subject 52 had a very low clearance compared with other subjects. When it was not included in the analysis, the clearance values of some subjects increased significantly, while the others decreased remarkably. We recognized this subject as an influential outlier. At first glance, subject 20 was not an outlier as it had a clearance value around the median. However, when it was deleted from the data set, the estimated clearance for most other subjects dropped accordingly, which indicated that the estimates were inflated when subject 20 was included in the previous analysis.

**Figure 7:**
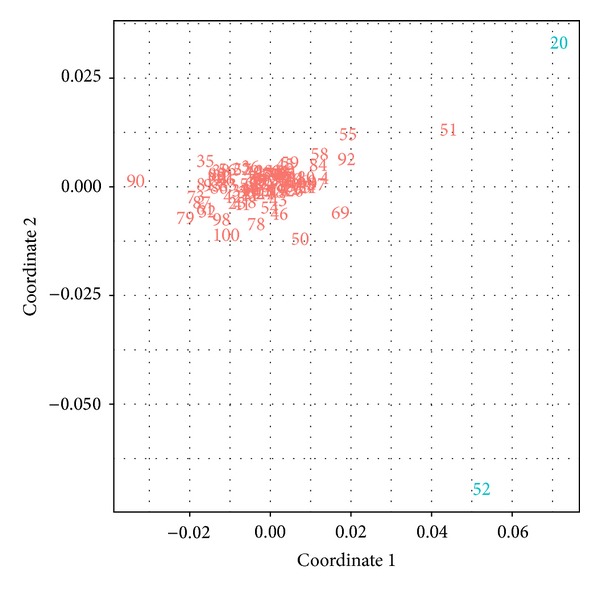
MDS plot for diagnosing case deletion runs for the clearance estimates. The IDs in the figure match the case deletion run ID, and it means that the patient with this ID was deleted. The plot indicates that subjects 52 and 20 were influential on clearance estimates.

The MDS plot is presented in Figure 7. In this analysis, subjects 52 and 20 were identified as the outliers suggesting that they may be influential on the clearance estimates.

### 3.5. Interactive Graphics

**Figure 8:**
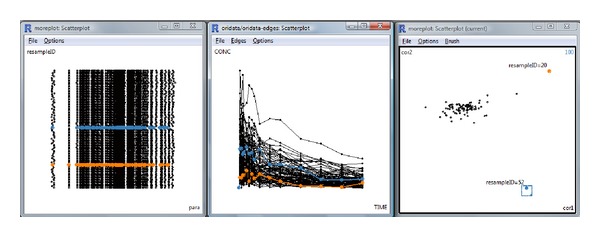
Interactive graphics for case deletion diagnostics: (left) parallel coordinate plot, (middle) time series plot for all patients, and (right) MDS plot. Two outliers with case deletion IDs 52 and 20 are brushed in the MDS plot, and the corresponding elements of the other two plots are colored accordingly.

Interactive graphics create an integrated framework to link the parallel coordinate plot, the MDS plot, and the plot of the subject concentration profiles. This technique provides opportunities to examine the model fit and inferred estimates from different perspectives based on independent algorithms. The result is shown in Figure 8 using GGobi package. The two outlying observations in the MDS plot (right) were marked in red and blue for subjects 20 and 52, respectively. All plots were linked by the sample ID (resample ID). Furthermore, the points representing these two samples were colored simultaneously in the parallel coordinate plot (the left panel), which was the approach that we used in the previous section to identify the influential subjects. In addition, these two subjects were also highlighted in the scatter plot of concentration time profiles of all subjects (the middle panel).

In fact, we can only see the difference in these two subjects from the others until the case deletion approach is performed. These two subjects were then deleted one by one to refit the model. The linked plots allow us to compare the information learned separately from individual plots and examine the other information about these subjects.

**Figure 9:**
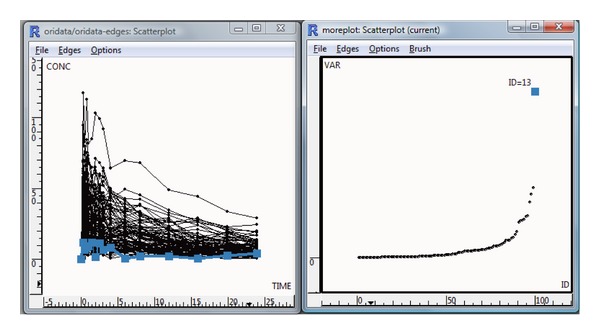
Interactive graphics for bootstrap statistics. In the left side plot variance of clearance estimates is plotted against subject ID. There is one subject with very large variance, which is brushed (blue). The plot is linked to the time series plot for all subjects (right).

Figure 9 describes a special case for examining the bootstrap statistics through interactive graphics. The left panel is a scatter plot of concentration versus time, and the right panel explores the variance of clearance versus the ordered ID. The subject having the largest variance in the clearance estimates was brushed with blue. Furthermore, this plot was linked to the left panel by the subject ID. Interestingly, we found that one subject (13) had very low concentration but had the highest variance of clearance.

## 4. Conclusions and Discussion

The main objective of this research was to develop effective and powerful visualization methods for resampling statistics that can be applied to diagnose population pharmacokinetic models. Two approaches were included: case deletion diagnostics and bootstrap. Case deletion diagnostics focuses on identifying the influential subjects, while bootstrap targets the variability of parameters and model robustness.

In this research, we tried to incorporate, for data analysis, the technique of interactive graphics, which has been available for several years. To our knowledge, this is the first attempt to implement interactive graphics in the pharmacokinetic field. We applied this technique to resampling data and demonstrated the feasibility and accessibility of the graphic approach related to case deletion diagnostics and bootstrap. With the support of interactive graphics, users can easily link all patients in various analyses and evaluate the results from a systematic perspective instead of some standalone parameter or multiple separated resources. Another contribution of this research is to provide new insight into model diagnostics with high-dimensional visualization approaches. By combining parallel coordinate plot and multidimensional scaling, we can transform high-dimensional data to low-dimensional data for visualization and clustering without losing the original information. However, there are still some limitations in our study. The tests were on a small data set that is publicly available. A larger pool of data sets will definitely contribute to the improvement of this new analysis strategy. Additionally, the approach requires several supporting software packages, which creates overheads before starting the analysis.

In conclusion, we developed several visualization methods to analyze multidimensional resampling data in the framework of interactive graphics. Several visualization techniques, including histogram, parallel coordinate plot, and multidimensional scaling, were implemented to explore the data structure and the hidden relations embedded in multiple resources. By combining static graphics with interactive graphics, we explored, screened, and investigated some outliers of these multidimensional data from an integrated and systematic perspective. This research is complementary to current approaches and presents a novel way to visualize and analyze the pharmacokinetic data.
